# Emergence of orthogonal hippocampal representations during spatial learning

**DOI:** 10.3758/s13420-025-00674-3

**Published:** 2025-05-01

**Authors:** Verner P. Bingman

**Affiliations:** Department of Psychology and J. P. Scott Center for Neuroscience, Mind and Behavior, Bowling Green State University, Bowling Green, OH USA

## Abstract

Sun et al. (2025) reveal the progressive, dynamic changes in the response properties of thousands of hippocampal neurons as mice learn a conditional discrimination while moving along a virtual linear track. At the end of training, separate orthogonalized ensemble codes, reflecting the properties of a state machine, capture the inherent structure of the task while dissociating the discrimination outcomes.

The discovery of the “place cell” in the rat hippocampus (O’Keefe & Dostrovsky, 1971) was a watershed moment in the history of cognitive neuroscience. That finding led to more than 20 years of research, seemingly dedicated to explaining practically all the variation in the firing rate of a place cell by the position of an animal in an environment, despite O’Keefe and Dostrovsky (1971) themselves pointing out that the firing rates of their place cells were influenced by factors other than position in space. Beginning in the 1990s, an expanding volume of data has revealed that the response properties of place cells capture much more than simply the position of a subject in space. Among other findings, the environmental (e.g., Wood et al., 2000) and psychological (e.g., Kelemen & Fenton, 2010) context of a task can strongly influence the firing patterns of hippocampal neurons, often revealing an orthogonal-like ensemble coding, a kind-of pattern separation (Leutgeb et al., 2007), of the same “spaces” in different contexts.

Employing a tour de force array of techniques, including the simultaneous recording of thousands of neurons using two-photon calcium imaging and training immobilized, and having transgenic mice walk on a rotating stage to move along a virtual linear track, Sun et al. (2025) offer unprecedented insight into the gradual emergence of two orthogonal hippocampal neuronal codes representing two distinct task-goal locations along the virtual linear track. The task was composed of two “contexts” in which the illumination of one of two indicator lights early along the linear track, the context cues, determined which of two visually distinct locations at subsequent positions along the linear track would deliver a water reward. At the beginning of training, CA1 hippocampal neurons fired relatively indiscriminately along the track. As training progressed and the subjects gradually learned the farther component of the conditional discrimination and later the nearer, the firing of hippocampal neurons gradually became increasingly specific to locations along the virtual track and the two contexts. The parallel progression in the behavioral learning of the task with the increased specificity in the response properties of hippocampal neurons was striking. Notable here is that locations associated with the indicator lights and the reward locations, the behaviorally more salient locations, were “decorrelated”—that is, associated with greater specificity in the response properties of hippocampal neurons, before the homogeneous grey spaces between these locations. At the end of training, the successful behavioral discrimination was accompanied by orthogonal hippocampal representations of the different contexts.

The paper then explores how various types of computational models capture both the behavioral and neuronal response property transitions observed in the test mice. Among the models, a closed-structured causal graph (CSCG) model best explained the orthogonalized state machine properties of the emergent hippocampal ensemble codes. As the authors write better than I can explain, “the ability of the CSCG model to recapitulate key features of hippocampal neural dynamics suggests that sequence-dependent extraction of latent states from potentially ambiguous or identical sensory inputs may represent a fundamental computational principle of hippocampal learning” (page 171, paragraph 1). Following from the modelling, the study carried out some informative probe training in which novel indicator stimuli were introduced into the task, requiring the mice to learn new conditional discriminations while the overall task structure remained the same. Not surprisingly, the mice learned the new discriminations more rapidly than the original. Of more interest, the spatial preferences of hippocampal neurons that responded to unchanged locations on the virtual maze remained relatively unchanged. By contrast, neuronal responding changed at the locations in the presence of the now new indicator lights and reward visual features. Viewing the hippocampus as a “state machine,”5 the data suggest that once learning has established a cognitive map for a particular task, or presumably and more broadly a spatial environment, it can be swiftly adapted to accommodate changes in the task properties of that space, facilitating new learning.

One of the more consequential implications of the paper is that it challenges the traditional approach of classifying hippocampal neurons as “types,” such as place cells, replacing that static view with a more dynamic characterization. The paper argues that the response properties of single hippocampal neurons should be considered along a continuum, with neurons behaving more like plastic “state cells.” In support of this alternative approach to classification is the observation of the changing response properties and functional roles of single hippocampal neurons as training progressed. Closing this section, the authors note that their findings support the idea that sparse orthogonal representations are a powerful mechanism for memory and intelligence.

The elegance of the experimental design and the significance of the results are justifiably laudable and far reaching, but there are aspects of the study that may limit how transformational it might be. Always a concern in a highly structured laboratory task is how well the results of Sun et al. (2025) would generalize to a task with different behavioral and spatial-environmental characteristics. Along these lines, acknowledging that the hippocampus and its importance for spatial cognition evolved under natural field conditions in freely moving animals, is it a conceit to assume that the progressive crystallization of orthogonalized hippocampal ensemble codes for different hippocampal spaces, as described in Sun et al. (2025), would occur under more ecologically relevant conditions? In other words, one is left wondering if the findings, especially the modelling results, may be more valuable for improving on how artificial intelligence systems work, and the engineers that develop them, rather than understanding the brain mechanisms that guide the complexity of naturally occurring spatial cognition and behavior.

Another confounding aspect of the study, a concern that generalizes to many studies investigating the relationship between the implementation of a cognitive map and hippocampal neuronal response properties, is the reliance on a behavioral task that is necessarily simple and whose solution would not require an intact hippocampus. First, the linear properties of the virtual maze render the navigational components of the task remote from anything that would engage a cognitive map, such as a rat finding a hidden platform in a water maze or a homing pigeon returning to its home loft by relying on familiar landscape features. Second, the stimuli of the conditional discrimination are feature based (i.e., different visual patterns) and spatial only in the sense that the correct goal location is associated with either the subsequent near (or sooner) or far (or later) visual feature stimulus. Neither of these procedural elements would generally be considered hippocampal dependent. A legitimate response to this concern is that even though an intact hippocampus would likely not be necessary to solve the task, the hippocampus is still “online” registering the spatial landscape and the conditional reward locations in that landscape. In any event, and as intimated above, these considerations render one less certain how the dynamic emergence of the still exciting orthogonal hippocampal representations described in Sun et al. (2025) would generalize to a context of true navigation and the *necessity* of a cognitive map to navigate to and recognize the position of a goal.

## Data Availability

Not applicable.
